# High density GaN/AlN quantum dots for deep UV LED with high quantum efficiency and temperature stability

**DOI:** 10.1038/srep05166

**Published:** 2014-06-05

**Authors:** Weihuang Yang, Jinchai Li, Yong Zhang, Po-Kai Huang, Tien-Chang Lu, Hao-Chung Kuo, Shuping Li, Xu Yang, Hangyang Chen, Dayi Liu, Junyong Kang

**Affiliations:** 1 Fujian Key Lab of Semiconductor Materials and Applications, Department of Physics, Xiamen University, Xiamen, 361005 (P. R. China); 2 Department of Electrical and Computer Engineering and Center for Optoelectronics, University of North Carolina at Charlotte, Charlotte, NC, 28223 (USA); 3 Department of Photonics and Institute of Electro-Optical Engineering, National Chiao-Tung University, Hsinchu, Taiwan, 30050 (China)

## Abstract

High internal efficiency and high temperature stability ultraviolet (UV) light-emitting diodes (LEDs) at 308 nm were achieved using high density (2.5 × 10^9^ cm^−2^) GaN/AlN quantum dots (QDs) grown by MOVPE. Photoluminescence shows the characteristic behaviors of QDs: nearly constant linewidth and emission energy, and linear dependence of the intensity with varying excitation power. More significantly, the radiative recombination was found to dominant from 15 to 300 K, with a high internal quantum efficiency of 62% even at room temperature.

AlGaN multiple-quantum-well (MQW) light-emitting diodes (LEDs) operating in the ultraviolet (UV) region are increasingly attracting attention due to a wide variety of potential applications, such as water purification, sterilization, medicine, biochemistry, white light solid-state lighting via phosphor excitation, and light source for high density optical recording. Although AlGaN MQW UV LEDs with emission wavelength shorter than 360 nm have been reported by several groups, the LED quantum efficiency drops dramatically with increasing Al content in order to decrease the wavelength, because of the difficulty in achieving high crystalline quality and doping efficiency in AlGaN at high Al content, and suppressing electron overflow from the QW^1^,^2^,^3^,^4^,^5^,^6^. Subsequently, the internal quantum efficiencies (IQE) of the AlGaN MQW LED is much lower than that of the InGaN blue LED, because of the high density typically found in the AlGaN MQW^7^,^8^,^9^,^10^. To reduce this problem, AlGaN MQWs have been grown on the bulk AlN, the nano-patterned sapphire substrate, or thick AlN layer epitaxied by high temperature metal-organic vapor phase epitaxy (MOVPE), in which the maximum external quantum efficiencies (EQE) of 10.4% is obtained^11^,^12^,^13^. However, the high cost of these methods limits their commercial use. Instead of the AlGaN MQW active regions, the GaN/AlN superlattice or quantum dots (QDs) structures have been proposed to be an alternative approach for UV-LEDs, in which the wavelength can be tuned from near-UV to deep-UV by decreasing the GaN layer thickness or QDs height^14^,^15^,^16^. More importantly, for the QDs structure, adding lateral carrier confinement to a vertical QW has been known for a long time to be able to suppress the nonradiative recombination loss through confining the carriers and preventing them from moving to dislocations^17^. Furthermore, single crystalline GaN QDs are likely to contain less point defects and more immune to the dislocations originated from the substrate. Therefore, one would expect that GaN quantum dots (QDs) can serve as efficient UV light emitters if they are properly confined by an AlN matrix^18^,^19^. Currently, self-assembled GaN/AlN QDs have been realized by both molecular beam epitaxy (MBE)^20^,^21^,^22^,^23^,^24^,^25^ and MOVPE^26^,^27^,^28^,^29^ in the Stranski-Krastanov (SK) growth mode, by either using anti-surfactants^30^ or taking advantage of the large strain caused by the large lattice mismatch between AlN and GaN. Because of the challenge in identifying appropriate barrier material for both injecting carriers and providing quantum confinement^31^, even though PL emissions of GaN/AlN heterostructure have been demonstrated with various wavelength^32^, only a few UV LEDs with GaN/AlN heterostructure as active region have been reported^33^,^34^,^35^,^36^,^37^.

In this work, we report the UV LED with room temperature (RT) emission near 300 nm, based on GaN/AlN QDs grown by MOVPE, with an estimated IQE as high as 62%. The LED structure was grown on *c*-plane sapphire substrates by MOVPE. The structures of the GaN/AlN QDs were investigated by scanning electron microscopy (SEM) and transmission electron microscopy (TEM). The vibrational mode of the GaN QDs was confirmed by the Raman scattering measurements. The emission of the GaN/AlN QDs was probed by spatially resolved cathodoluminescence (CL) as well as macroscopic EL. The peak position and width of the EL band, an ensemble of the emission of different QDs, is found nearly independent of temperature, characteristic of the QD emission. The IQE and recombination dynamics were analyzed by temperature-dependent PL and time-resolved photoluminescence (TRPL) between 15 K and 300 K.

## Results

**Figure 1:**
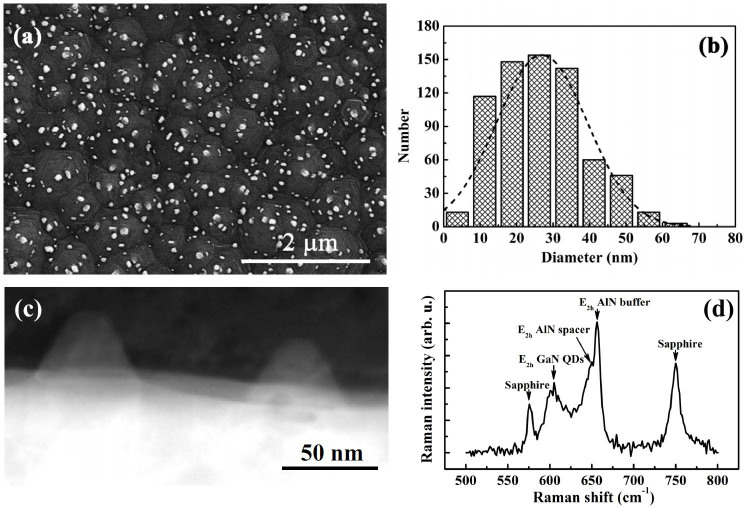
(a) SEM, (b) size distribution, (c) cross-sectional TEM image and (d) Raman spectrum of the self-assembled GaN/AlN QDs grown on *n*- Al_0.7_Ga_0.3_N, respectively.

The surface morphology of the sample after growth was imaged by SEM. As shown in Figure 1a, many isolated dot-liked nanostructures with density of about 2.5 × 10^9^ cm^−2^ are self-assembly formed on the hexagonal hillocks of the underlying *n*-type Al_0.7_Ga_0.3_N layer. The sizes of the nanostructures are mostly in the range of 10–35 nm and centered at around 25 nm, as shown in Figure 1b. Moreover, cross-sectional TEM investigations were carried out for such nanostructures. It can be seen in Figure 1c that the nanostructure appears as hexagonal truncated pyramids. Since the detailed internal structure cannot be recognized in the TEM image, the phonons in the sample was analyzed using Raman scattering to gain a better understanding of these structures. Figure 1d displays the recorded Raman spectrum. Two peaks, related to the sapphire substrate (around 576 cm^−1^ and 750 cm^−1^) can be observed. Also, there is a peak centered at 657 cm^−1^, which can be assigned to the E_2h_ phonon mode of high-temperature AlN layer grown on the sapphire with a little compressive strain. In particular, another two peaks, one broader peak centered at around 604 cm^−1^ and one shoulder peak around at 649 cm^−1^, appear in the spectrum. These two Raman frequencies are very close to the reported values of E_2h_ phonon mode of the GaN QDs and the AlN spacer, respectively^38^,^39^. Since in the investigated sample, the GaN was deposited first on the hexagonal hillocks Al_0.7_Ga_0.3_N layer, GaN QDs maybe self-assembly formed due to the lattice-mismatch between GaN and Al_0.7_Ga_0.3_N. Then, the AlN barrier-layer was grown, and the followed GaN will keep on the island-patterns due to easier release of elastic energy. In light of this, it can be deduced that the nanostructures investigated in both SEM and TEM images may contain multiple stacked plans of GaN/AlN QDs.

**Figure 2:**
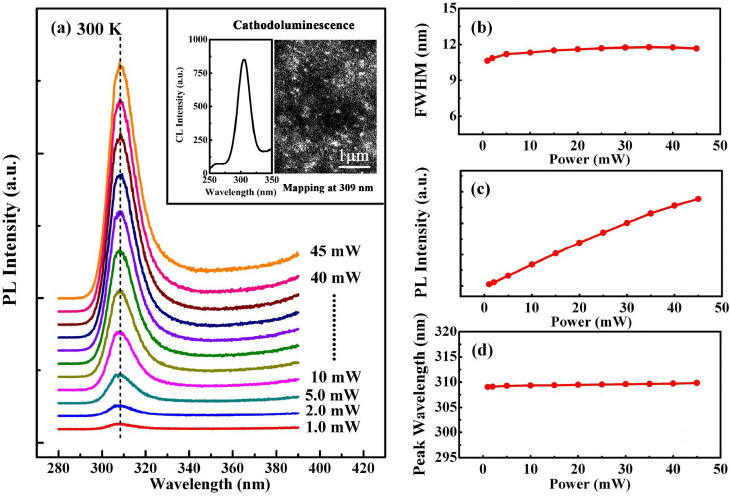
Power–dependent PL taken at room temperature. (a) Power-dependent PL spectra of the GaN/AlN QDs at 300 K. Inset shows the CL spectrum and monochromatic CL mapping at wavelength of 309 nm at 300 K. (b) Spectral width, (c) integrated intensity and (d) peak wavelength of the PL spectrum as a function of excitation power, respectively.

Furthermore, the emission of the GaN/AlN dots was characterized by PL and CL in UV region. A dominant emission band is centered around 309 nm, which is 0.595 eV higher than the band gap of unstrained bulk GaN, even when the power of the pulsed excitation increases from 1.0 to 45 mW, as illustrated in Figure 2a. The 309 nm emission was found to come from the GaN/AlN dots by carrying out the monochromatic CL mapping, as shown in the inset of Figure 2a. The larger blueshift of the emission energy with respect to the band gap of bulk GaN can be attributed to the small QD height. Similar emission wavelength of 310 nm in PL has been reported previously for 3 monolayer thick GaN QDs embedded in AlN barriers, where the AlN layer remains continued^32^. This indicates that the vertical size of GaN QDs exiting in the nanostructures in our sample is less than 3 monolayer.

The excitation power dependence of full width at half maximum (FWHM), integrated intensity and peak wavelength of the 309 nm emission band were determined by fitting the PL spectra with a Gaussian function. As shown in Figure 2b, over the excitation power range of 1.0 to 45 mW, FWHM is almost constant with a small value of 11.8 nm (153 meV), even though the QD lateral sizes fluctuate substantially. The integrated PL intensity changes over more than two orders of magnitude and depends almost linearly on the excitation power without saturation even when the excitation power reaches 45 mW. This implies that the QD levels have a relatively high density of states (DOS), large subband separations, and weak nonradiative recombination. Moreover, it is worthy noting that, when the excitation power increases from 1.0 to 45 mW, the wavelength of the emission exhibits no blue shift, in contrast to what is typically observed in the wurtzite nitride-based QW as well as the larger GaN QDs with giant polarization field.

**Figure 3:**
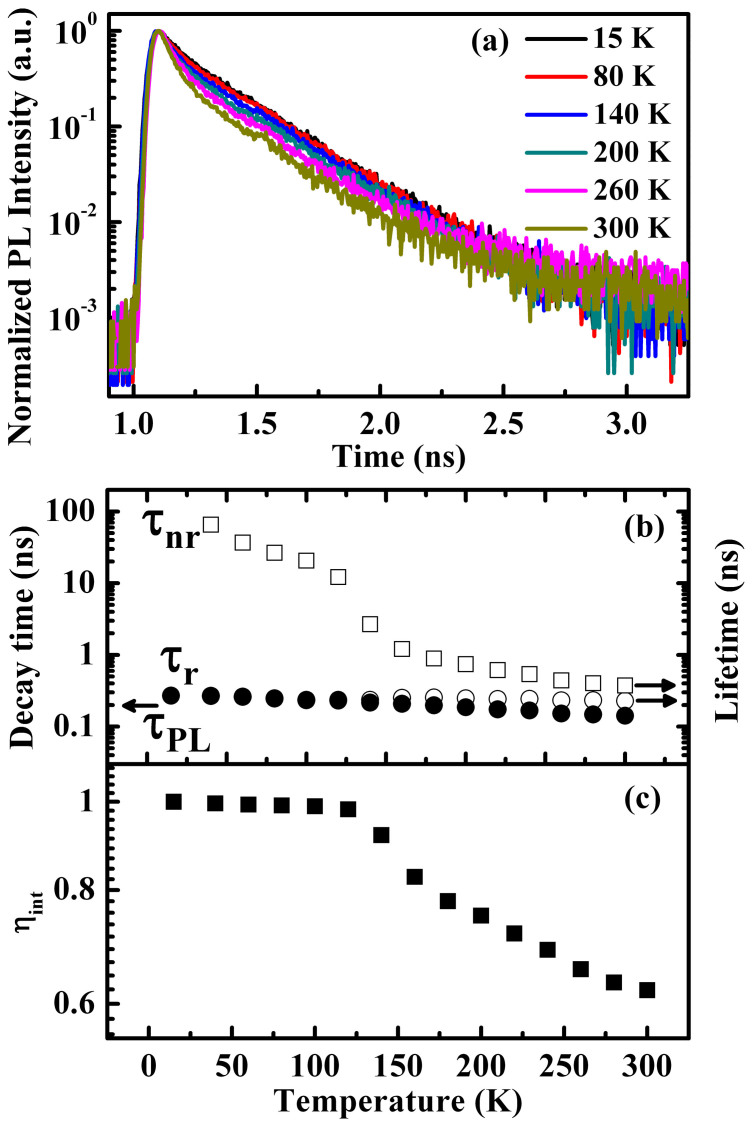
Temperature–dependent TRPL taken in temperatures ranging from 15 K to 300 K. (a) TRPL spectra for GaN/AlN QDs as a function of temperature (15–300 K). (b) Temperature-dependent decay time (closed circles), radiative (open circles) and nonradiative lifetimes (open squares). (c) Temperature-dependent PL efficiency (closed squares).

To get more insight into the recombination dynamics of GaN/AlN QDs and assess the IQE (η_int_), the temperature-dependent time-resolved PL (TRPL) and temperature-dependent PL measurements were carried out in 15–300 K range, as illustrated in Figure 3a. Although the decay is shorter in higher temperature, all of them are fast in the order of sub-nanoseconds over the temperature in range of 15–300 K. To simplify the analysis, we take the 1/*e* decay times as the PL decay time (τ_PL_), although the decay might not be exactly single exponential. The temperature dependence of τ_PL_ is plotted in Figure 3b. The τ_PL_ merely changes from 0.27 to 0.14 ns as the temperature increases from 15 to 300 K. Note that the PL decay time is related to the radiative (τ_r_) and nonradiative recombination (τ_nr_) times by 1/τ_PL_ = 1/τ_r_ + 1/τ_nr_. Additionally, η_int_ can be written as a fraction of radiative rate over the sum of radiative and nonradiative rates, i.e., η_int_ = (1 + τ_r_/τ_nr_)^−1^ 40. Hence, by solving these two equations, both τ_r_ and τ_nr_ can be obtained. In general, η_int_ can be approximated as the spectrally integrated PL intensity at given temperature T over that at low temperature (Figure 3c), assuming there is no nonradiative recombination at low temperature^8^. On the basis of these, the temperature dependences of the radiative and nonradiative recombination times are illustrated in Figure 3b. The radiative recombination time remains constant from 15 to 300 K, confirming the efficient localization of the carriers within the GaN QDs. Meanwhile, the nonradiative recombination time decreases monotonically with increasing temperature and is always longer than the radiative recombination time. This demonstrates that the radiative recombination is dominant at any temperature and the nonradiative recombination is well suppressed. As a result, the estimated IQE of our GaN QD structure reaches as high as 62%, which is much higher than that of UV LEDs in similar wavelength region but using AlGaN QWs as the active layer^7^,^8^. This demonstrates the intrinsic advantage of the QD structure in promoting the radiative recombination and in the mean time suppressing the nonradiative recombination loss.

**Figure 4:**
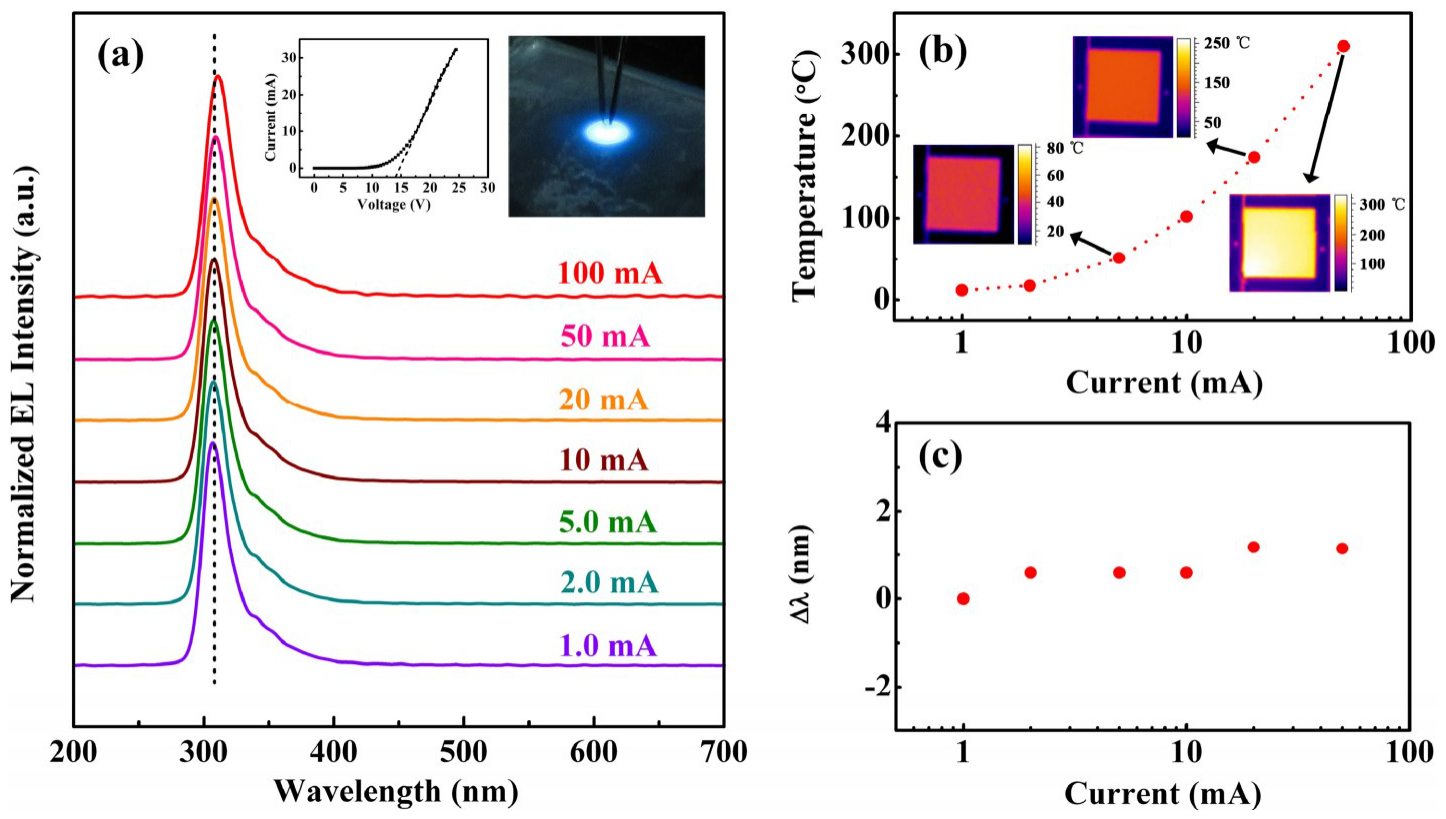
Electrical characteristics of GaN/AlN QDs UV LED. (a) EL spectra from the GaN/AlN QDs UV LED with varying DC current at RT. Insets depict the current-voltage characteristics of the fabricated GaN/AlN-QD UV-LED chip and visible blue emission from blue phosphors excited by the UV emission. (b) The temperature distributions of the UV-LED chip under different injection currents. (c) Wavelength shift of the UV-LED chip in the 1.0–100 mA range with respect to 1.0 mA.

Finally, for the device fabrication, an Al_0.8_Ga_0.2_N electron blocking layer and a *p*-type layer consisting of our designed thirteen-period Mg and Si δ-doped superlattices^41^ were deposited to finish the growth of a full UV LED structure. After that, the LED chips were fabricated in a mesa size of 1000 μm × 1000 μm by using the regular chip process with photolithograph, dry etching, and metal evaporation. In order to improve heat dissipation and light extraction, the devices were flip-chip mounted on a Si submount and packaged in TO-5. The EL measurements were carried out under DC bias with current in the range of 1–100 mA at 300 K, as shown in Figure 4a. It can be seen that the main EL peak related to GaN QDs is around 308 nm which agrees well with the PL peak. And the emission mechanism of ~350 nm peak on the shoulder of the main peak is unclear now, but may be introduced during the chip fabrication process, since it cannot be observed in PL spectra. The inset of Figure 4a depicts the current-voltage (I-V) characteristics of a typical fabricated GaN/AlN QD UV-LED chip. The device exhibits a turn-on voltage of about 14 V. The other inset shows bright blue emission from coated blue phosphors excited by the UV emission of the device through the transparent structure. This result is also an indicator that the UV emission is rather strong. Furthermore, the temperature distributions across the chip surface under different injection currents were characterized by a thermal infrared imager. As seen from Figure 4b, the temperature of the chip increases significantly with increasing injection current. The amount of the increment is as much as 293°C when the injection current increases from 1.0 mA to 50 mA. This could be resulted from inefficient light extraction and relatively high resistivity of high Al content AlGaN. Even though suffering from the large variation of temperature, the EL peak position exhibits only a small redshift of Δλ ~ 0.56 nm (7.8 meV) which is much smaller than the band gap shrinkage of bulk GaN (105 meV), as illustrated in Figure 4c. Thus, the QD structure is also able to offer much better thermal stability of the emission wavelength. Over all, we have demonstrated UV LEDs using GaN/AlN QDs with high IQE and wavelength stability.

## Discussion

In conclusion, the GaN/AlN QDs with the density of 2.5 × 10^9^ cm^−2^ have been demonstrated on *n*-type Al_0.7_Ga_0.3_N by MOVPE. The PL and CL measurements confirm that the GaN/AlN QDs emit light as short as 309 nm, which is 0.595 eV higher than the band gap of unstrained bulk GaN. More importantly, the QD structure possesses highly desirable characters for an efficient UV LED: (1) radiative recombination dominates from 15 to 300 K, leading to a high room temperature IQE greater than 50%, (2) small and nearly constant FWHM (153 meV) and emission wavelength, and (3) linear dependence of the PL intensity and high emission wavelength stability with varying excitation power. The fabricated LEDs emit very efficiently at 308 nm, practically the same wavelength as the PL, showing high temperature stability in both emission wavelength and line width. This work demonstrates the intrinsic advantage of the QD approach for the deep UV LED, that is, the QD structure can effectively confine and accumulate the carriers, and also to suppress the nonradiative recombination.

## Methods

### Fabrication

All the samples used in this work were grown on (0001) sapphire substrates by MOVPE in a vertical reactor (Thomas Swan 3 × 2″ CCS). During the growth, trimethylgallium (TMGa), trimethylaluminum (TMAl), and ammonia (NH_3_) were used as the precursors, with high purity H_2_ as the carrier gas. Biscyclopentadienyl magnesium (Cp_2_Mg) and silane (SiH_4_) were used as the *p*- and *n*-type doping sources, respectively. It is known that pulsed atomic layer epitaxy (PALE) is an effective method to grow AlN or high Al content AlGaN^42^,^43^,^44^. We have successfully employed this PALE method to grow AlN buffer layer for the further growth of high quality AlN and AlGaN with high Al content, which leads to the successful fabrication of deep UV LEDs^45^,^46^. The structure initiated with a 20-nm-thick AlN buffer layer via PALE, followed by a 230 nm high-temperature AlN. Subsequently, a 1.35-μm-thick *n*-type Al_0.7_Ga_0.3_N layer was grown, followed by the epitaxy of five-pair GaN/AlN double-layers consisting of 1-nm-thick GaN and 10-nm-thick AlN. A growth interruption of ten seconds was introduced between the growth of GaN and AlN to prevent the Ga-Al inter-diffusion.

### Measurements

The surface morphology of the as-grown wafer was investigated by SEM (LEO 1530 FEG at 20 kV) and the structural properties was characterized by TEM (Tecnai G2 F20 S-Twin). The Raman measurement was performed at room temperature along the wurtzite *c*-axis of the sample by confocal Raman spectroscopy (Renishaw inVia Raman Microscope) with a 532 nm laser excitation source. CL spectrum and the monochromatic scanning CL image at wavelength of 309 nm were measured at 300 K, using a Gatan monoCL equipped on a JEOL JSM-7000F FE-SEM. The power-dependent PL at 300 K and temperature-dependent PL and TRPL from 15 to 300 K, respectively, were excited by a frequency tripled Ti: sapphire laser at 266 nm with pulse width of 200 fs and repetition rate of 76 MHz. The luminescence spectrum was dispersed by a 0.55 m monochromator with the 2400 grooves/mm grating and detected by a high sensitivity photomultiplier tube for ultraviolet-visible wavelength. The injection-current dependent EL measurements for the UV LEDs with the GaN/AlN QDs as the active region were carried out under DC bias in the range of 1–100 mA. And the temperature distributions of the chip on the device under different injection currents were characterized by a thermal infrared imager (Mission Research-N2).

## Author Contributions

W.H.Y., X.Y., H.Y.C. and D.Y.L. fabricated the sample. W.H.Y., J.C. Li and P.K.H. carried out the measurements. W.H.Y., J.C.L., Y.Z., T.C.L., H.C.K., S.P.L. and J.Y.K. participated in the final data analysis. W.H.Y., J.C.L., Y.Z. and J.Y.K. drafted the manuscript. J.C.L. and J.Y.K. supervised the study. All the authors reviewed the manuscript.
